# Radiographic Findings of Inflammatory Arthritis and Mimics in the Hands

**DOI:** 10.3390/diagnostics12092134

**Published:** 2022-09-02

**Authors:** Fatemeh Ezzati, Parham Pezeshk

**Affiliations:** 1 Division of Rheumatic Disease, Department of Internal Medicine, UT Southwestern Medical Center, Dallas, TX 75390, USA; 2 Division of Musculoskeletal Radiology, Department of Radiology, UT Southwestern Medical Center, Dallas, TX 75390, USA

**Keywords:** imaging, radiographs, inflammatory arthritis

## Abstract

Clinical presentation could be challenging in patients with arthralgia, and imaging plays an important role in the evaluation of these patients to make the diagnosis or narrow the differential diagnosis. Radiography of the hands is a commonly available imaging modality that can provide crucial information with regard to the pattern and pathology of the involved joints. It is important that radiologists and rheumatologists are familiar with the imaging findings of different rheumatic diseases to make the diagnosis in the early stages of disease to initiate treatment.

## 1. Introduction

Hand radiographs are frequently ordered as the first imaging modality in the assessment of patients presenting with peripheral arthritis. They can provide invaluable information about the bones, joints, mineralization, soft tissues and the distribution of abnormalities. Given the wide spectrum of rheumatic diseases, it might be challenging to make the diagnosis solely based on the clinical findings and imaging plays an important role in narrowing the differential diagnosis. Having the knowledge of the common radiographic manifestations of inflammatory arthritis is of paramount importance for clinicians and radiologists to diagnose the underlying disease in early stages of disease in order to start treatment. The purpose of the article is to review the key radiographic findings of common rheumatic diseases in the hands.

## 2. Radiographic Views

**Figure 1:**
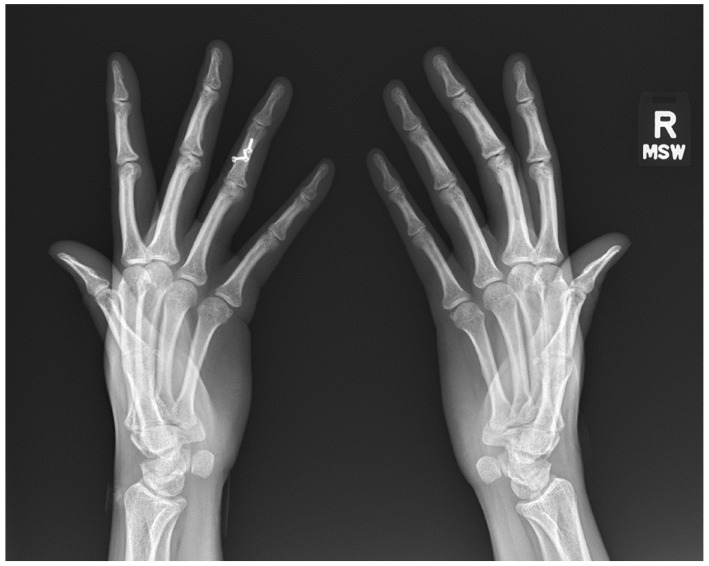
Norgaard (ball-catcher) view.

The standard radiographic views of the hands for evaluation of rheumatic disease include a posteroanterior (PA), an oblique, and a lateral view. Norgaard view (aka. ball-catcher view) can be obtained as an alternative to oblique views to evaluate the joints in an oblique angle [1]. The PA view is useful to assess soft tissues, alignment, mineralization, erosions and joint spaces. The oblique or Norgaard views are more sensitive for evaluation of erosions in the corners of the joints that may be obscured on the PA and lateral views (Figure 1). In Norgaard view, the patient is asked to position the hands similar to holding a ball with the palms facing up. This maneuver flexes the joints and exposes the corners of the joints to the X-ray beams.

## 3. Interpretation of the Hand Radiographs

To ensure a thorough assessment of hand radiographs, the mnemonics of “ABCDES” can be followed. In evaluating the alignment (A), attention should be paid to subluxation, dislocation, angulation and deviation of the joints. Bones (B) should be evaluated for trabecular pattern, destruction, and osseous lesions. Joint spaces should be evaluated for cartilage loss (C) and joint space narrowing, and bones need to be assessed for bone density and demineralization (D). The evaluation of erosions (E) as well as their pattern and distribution is also an important step in the assessment of patients with inflammatory arthritis. Soft tissue (S) swelling and calcifications can add crucial information to narrow the differential diagnosis. Upon gathering all the information, the clinician will have a better insight into the nature of underlying disease in the light of clinical findings. Radiographs of the hands can be used for evaluation of new patients as well as monitoring the progression of known disease.

The purpose of this note is to review the main radiographic findings of common rheumatic diseases and their mimics in the hands. Table 1 summarizes the radiographic findings that will be discussed in further details.

## 4. Rheumatoid Arthritis

Rheumatoid arthritis (RA) is a daily disease seen in rheumatology clinics. It predominantly involves the appendicular skeleton. The axial skeleton is mostly spared except for the cervical spine where instability can occur and can be carefully assessed with cervical spine radiographs in flexion and extension. Radiographs may show erosion at the C1-C2 level with destruction of the transverse ligament that can result in atlantoaxial subluxation [2].

**Figure 2:**
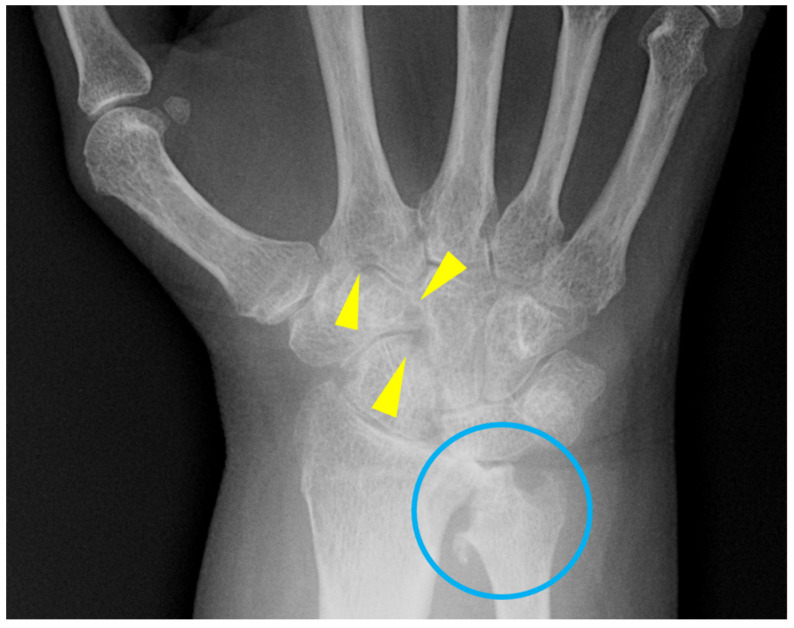
57-year-old female with rheumatoid arthritis. Moderate demineralization with scattered erosions in the carpal bones (yellow arrowheads) as well as distal radius and ulna (blue circle) are noted.

**Figure 3:**
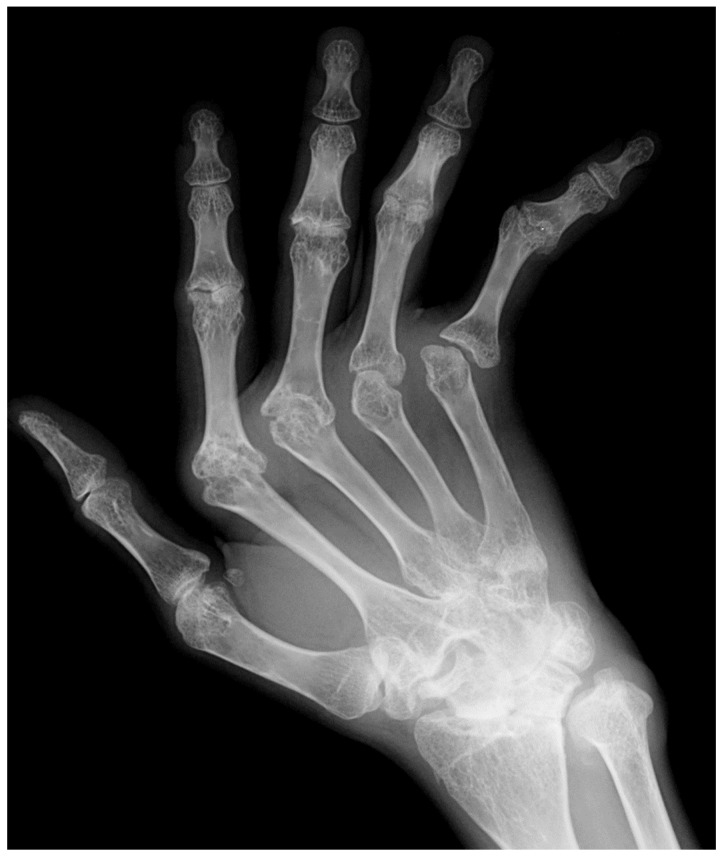
60-year-old female with long-standing rheumatoid arthritis. Hand radiographs shows erosive changes in the PIP and MCP joints as well as the wrist. Ulnar subluxation of the fingers at MCP joints is also present. Note the distal interphalangeal joints are spared.

**Figure 4:**
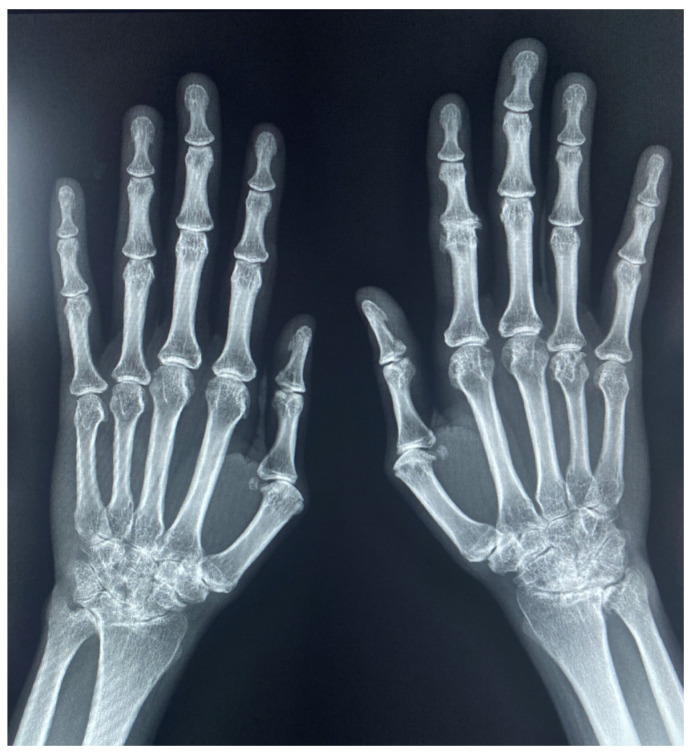
Frontal view of the hand in a 40-year-old female with rheumatoid arthritis. Severe pancarpal joint space loss is present as well as juxta-articular demineralization. Distal interphalangeal joints are spared.

In the early stage of the disease, synovitis and inflammation of the joints result in fusiform and symmetric juxta-articular soft tissue swelling. Due to hyperemia of the synovium and soft tissues in the setting of inflammation, bone resorption and peri-articular demineralization develops. The bare areas of the bones, where no covering cartilage exists, are exposed to the inflamed synovium and can be eroded [3,4]. These marginal erosions are the first erosive changes seen through the course of the disease and happen before joint space loss. Early erosive changes are appreciated as subtle loss and discontinuity in the cortex in the metacarpal heads as well as the radial base of the proximal phalanges at the metacarpophalangeal joints [4]. In the wrist, erosive changes are typically seen in the ulnar styloid, waists of the scaphoid and hamate, as well as the fifth carpometacarpal joint (Figure 2). As the cartilage destruction continues, symmetric and uniform loss of joint spaces develops and the underlying bone becomes exposed and eroded, which manifests as subchondral erosions. In contrast to osteoarthritis, the joint space loss in the RA is symmetric and mechanical reactive bony changes such as osteophytosis, sclerosis, or cystic changes are not appreciated as the initial manifestations until a later stage of the disease when secondary osteoarthritis develops. Rheumatoid arthritis does not typically involve the distal interphalangeal joints (DIPs) [5] and other possibilities should be entertained when DIP joints are involved (Figure 3 and Figure 4). Radiographs have low sensitivity in detection of bone erosions in the early stage of rheumatoid arthritis and some studies suggest that such findings do not predict the development of inflammatory arthritis in at-risk individuals with positive CCP (cyclic citrullinated peptide) [6]. Ultrasound and MRI with contrast are more sensitive imaging modalities to assess inflammation in the early phase when radiographs are normal.

Scoring radiographic findings in rheumatoid arthritis is used as an outcome measure to help estimate the progress of disease. Findings such as erosions, joint-space narrowing, demineralization, malalignment, soft tissue swelling, subluxation, ankylosis and cysts are used in different methods of scoring. The most commonly used scoring methods include van der Heijde-modified Sharp, Simple Erosion Narrowing Score (SENS) [7].

**Figure 5:**
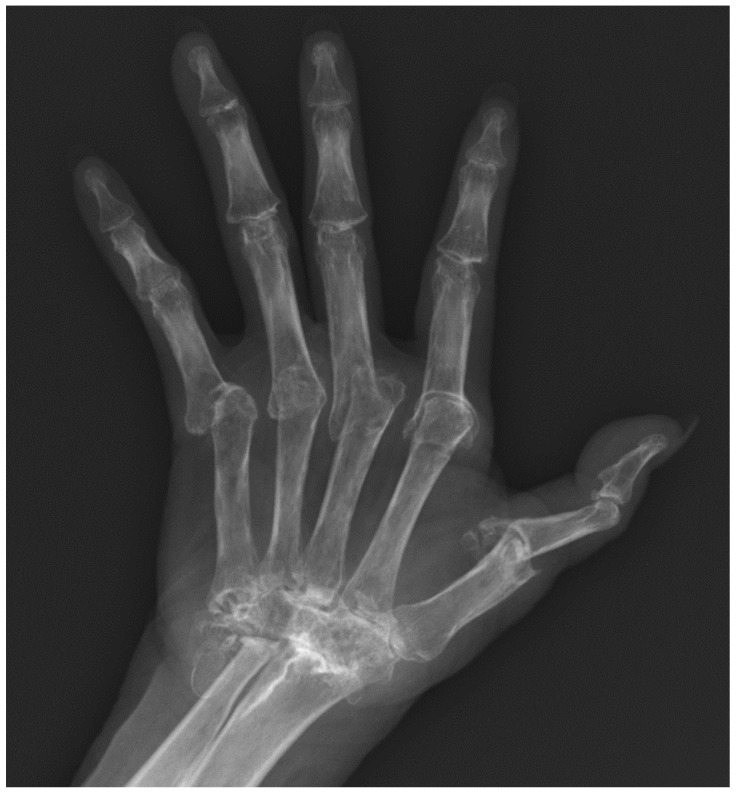
59-year-old female rheumatoid arthritis. Hand radiograph shows extensive erosive changes and osseous loss in the wrist and metacarpophalangeal joints with telescoping and ulnar deviation at the MCP joints. Severe demineralization is also present. Findings represent arthritis mutilans which also can be seen in psoriatic arthritis.

Inflammation of the tendons and ligaments around the joints of the hands results in malalignment and deformity of the joints. These deformities include subluxation of the MCP joints with ulnar and palmar subluxation of the proximal phalanges as well as boutonniere and swan-neck deformities of the distal phalanges. In the later stages of the disease, diffuse demineralization and ankylosis of the joints can also occur. Deformity of the hand can eventually present as arthritis mutilans (Figure 5). 

## 5. Psoriatic Arthritis (PsA)

**Figure 6:**
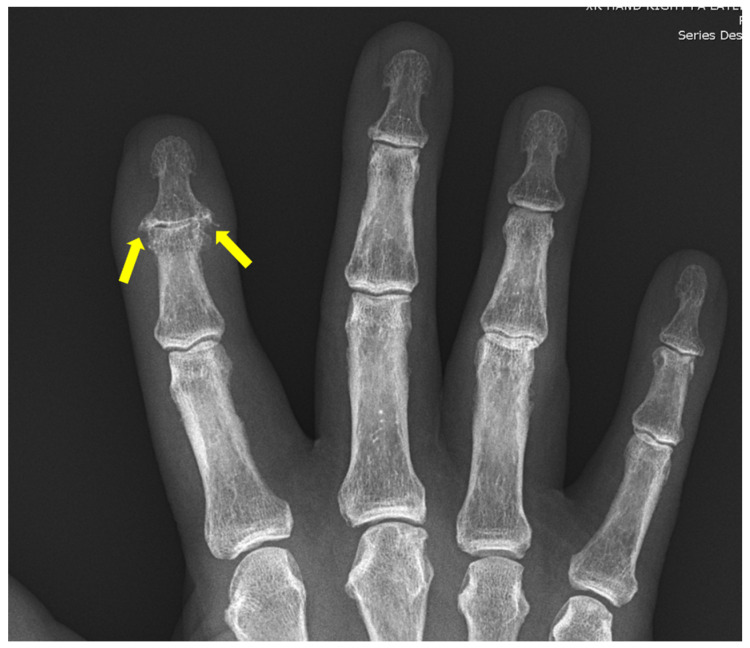
79-year-old male with psoriatic arthritis. Hand radiographs demonstrate diffuse soft-tissue swelling in the index finger (sausage finger). Small marginal erosions are present in the DIP joint along with mild fluffy periosteal reaction.

**Figure 7:**
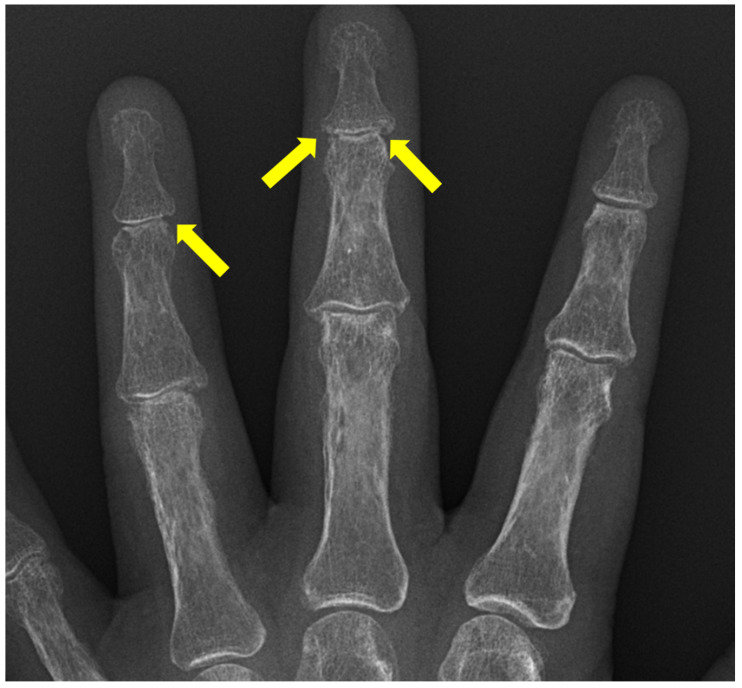
59-year-old female with psoriatic arthritis. Hand radiographs show marginal erosions in the distal interphalangeal joints of the index and middle fingers (yellow arrows). Mild periosteal reaction is seen at the middle finger DIP joints.

Psoriasis arthritis coincide or develop after skin changes. In 15% of cases, arthritis can precede skin changes by 2 years [8] and imaging can play an important role in diagnosis in this timeframe. Hands are the most commonly involved joints in psoriatic arthritis and interphalangeal joints are the predominantly affected [9]. Fusiform periarticular soft tissue swelling can be seen and in approximately 25% of the patients soft-tissue swelling extends beyond the joints and diffusely involves the entire digit resulting in dactylitis that is commonly known as hot-dog or sausage-digits (Figure 6). PsA mainly involves the distal and proximal interphalangeal joints [9]. Normal mineralization is usually maintained through the course of the disease, although early transient juxta-articular demineralization can be seen. Initially, marginal erosions develop which over time progress to central erosions with “pencil-in-cup” deformity. Arthritis mutilans occurs in about 5% of the patients and manifests as destruction of the joints and clinical “telescoping” of the joints where fingers can be pulled back to normal length. Acro-osteolysis is often a prominent feature of psoriatic arthritis [10]. Bony proliferation is the main finding in psoriatic arthritis, which, along with DIP involvement, differentiates it from the rheumatoid arthritis. Erosions in psoriasis are predominantly marginal, which can help differentiate it from central erosion in erosive osteoarthritis (Figure 6 and Figure 7). Marginal erosions with periostitis result in a mouse-ear appearance known as the “Mickey mouse” sign.

## 6. Erosive Osteoarthritis

**Figure 8:**
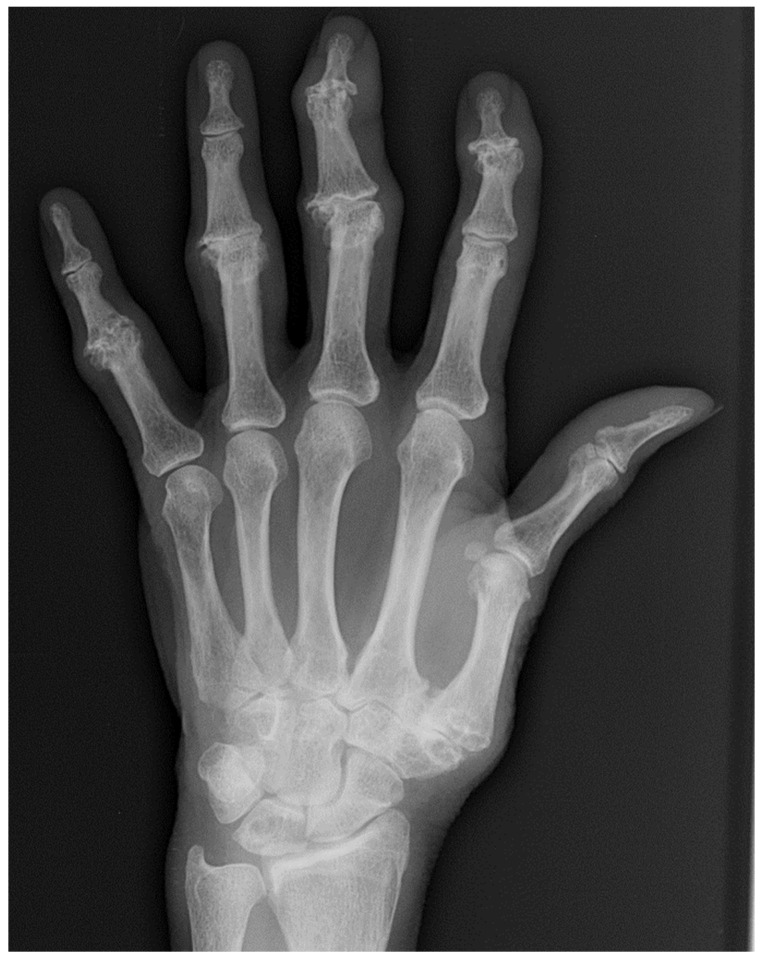
76-year-old female with erosive arthritis. Severe osteoarthritis of most of the distal and proximal interphalangeal joints with central erosions and seagull appearance.

**Figure 9:**
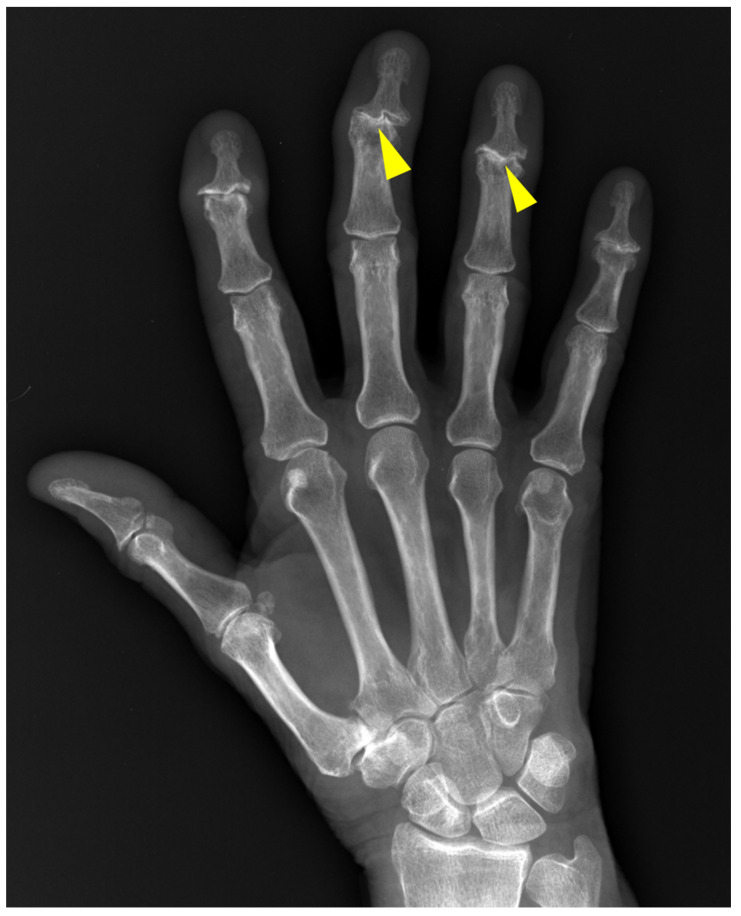
50-year-old female with erosive arthritis. Severe osteoarthritis of DIP joints with central erosions and seagull appearance (yellow arrowheads).

Erosive osteoarthritis (EOA) is a type of osteoarthritis with a strong inflammatory component. It occurs most commonly in females over the age of 60 and usually involves the interphalangeal joints in the hands. Radiographic findings typically include a combination of bony proliferation and erosions [11]. Distal interphalangeal joints are mostly affected and metacarpal (MCP) joints are usually spared. The hallmark of EOA is central erosions in the interphalangeal joints that result in gull-wing or seagull appearance [12] (Figure 8 and Figure 9). The first carpometacarpal joint is also frequently involved. In later stages, ankylosis of the joints might occur as well [13]. The main differential diagnosis of EOA includes classic osteoarthritis, rheumatoid arthritis, and psoriatic arthritis. Joint erosions and interphalangeal ankylosis are absent in classic osteoarthritis. While osteophyte formation is a consistent phenomenon in EOA, it develops secondary to degenerative changes in RA and PsA and is almost never seen as a primary feature in these two entities [11]. Unlike EOA, the DIP joints are spared in adult-onset RA; however, DIPs may be involved in juvenile idiopathic arthritis. In addition, RA demonstrates juxta-articular demineralization, a feature that is usually absent in EOA. Both EOA and PsA involve the DIP joints, but the erosions of PsA are marginal simiar to RA rather than central in EOA. PsA involves the joints asymmetrically unlike EOA, which almost invariably has a symmetric distribution [14]. PsA demonstrates additional features such as skin lesions, sacroiliitis, and enthesophytes followed by fluffy periostitis and sometimes bone erosions [15]. The DIP erosions in PsA has a “mouse-ear” appearance rather a “gull-wing” configuration of EOA [14]. Acro-osteolysis, pencil-in-cup deformity, and arthritis mutilans are absent in EOA [11].

## 7. Calcium Pyrophosphate Dehydrate Arthropathy (CPPD)

Calcium pyrophosphate dihydrate (CPPD) crystal deposition disease, also known as pseudogout, is the most common crystal arthropathy and is typically seen in the middle-aged and elderly population. Chondrocalcinosis is the deposition of CPPD crystals in the cartilage. CPPD arthropathy can present clinically as acute and chronic arthritis or destructive arthropathy [16]. It can be primary or secondary and the secondary form is the consequence of metabolic diseases such as hyperparathyroidism and hemochromatosis [17]. Chondrocalcinosis most frequently involve the knees, symphysis pubis and wrists. 

**Figure 10:**
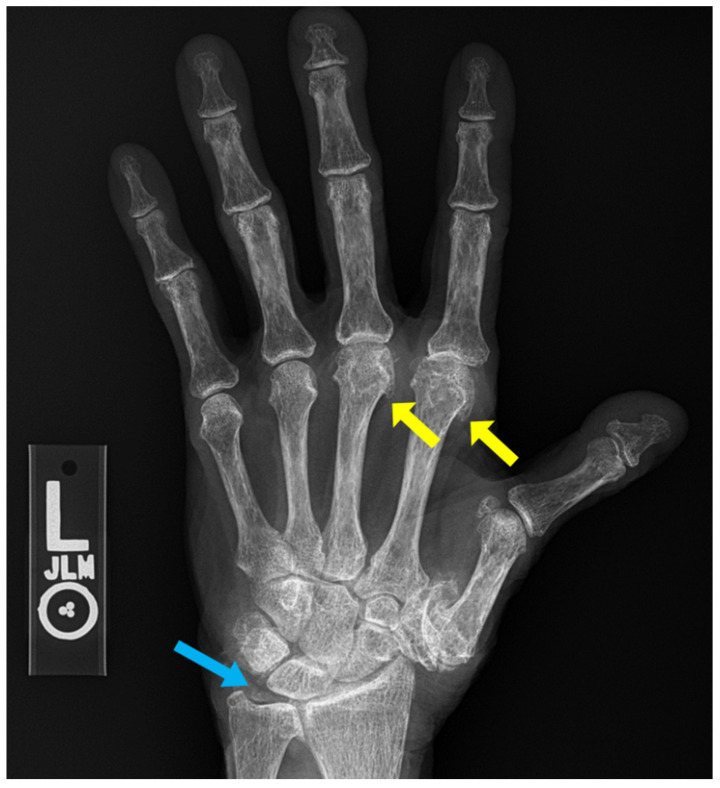
80-year-old male with CPPD arthropathy. Severe first carpometacarpal osteoarthritis, chondrocalcinosis (blue arrow), degenerative changes and hook osteophytes in the second and third metacarpals (yellow arrows) are findings to lead to the diagnosis.

Two main radiographic manifestations of CPPD include calcifications and arthropathy [17]. In the hands and wrists, chondrocalcinosis is mostly seen in the triangular fibrocartilage and cartilage adjacent to scaphoid, lunate and metacarpophalangeal joints. Bone mineralization is normal; however, it can be seen due to other etiologies such as aging. Joint-space loss is noted with subchondral new bone formation. CPPD arthropathy resembles osteoarthritis but in an atypical distribution. Common radiographic findings in CPPD include joint-space narrowing, subchondral cyst formation and osteophyte formation. Subchondral cysts are more significant than in primary osteoarthritis and could be the dominant picture in radiography. In the hands, CPPD arthropathy is usually confined to the metacarpophalangeal (MCP) joints, mostly involving 1st to 3rd MCPs, and spares interphalangeal (IP) joints. Beak-like osseous projections, also known as “hook osteophytes” could be seen in the 2nd and 3rd metacarpal head (Figure 10). In the wrist, CPPD arthropathy most commonly affects the radiocarpal joint and ligaments, including the lunotriquetral ligament, scapholunate ligament and triagnulat fibrocartilage complex (TFCC). Involvement and rupture of the scapholunate ligament may result in scapholunate advance collapse (SLAC) wrist [18].

**Figure 11:**
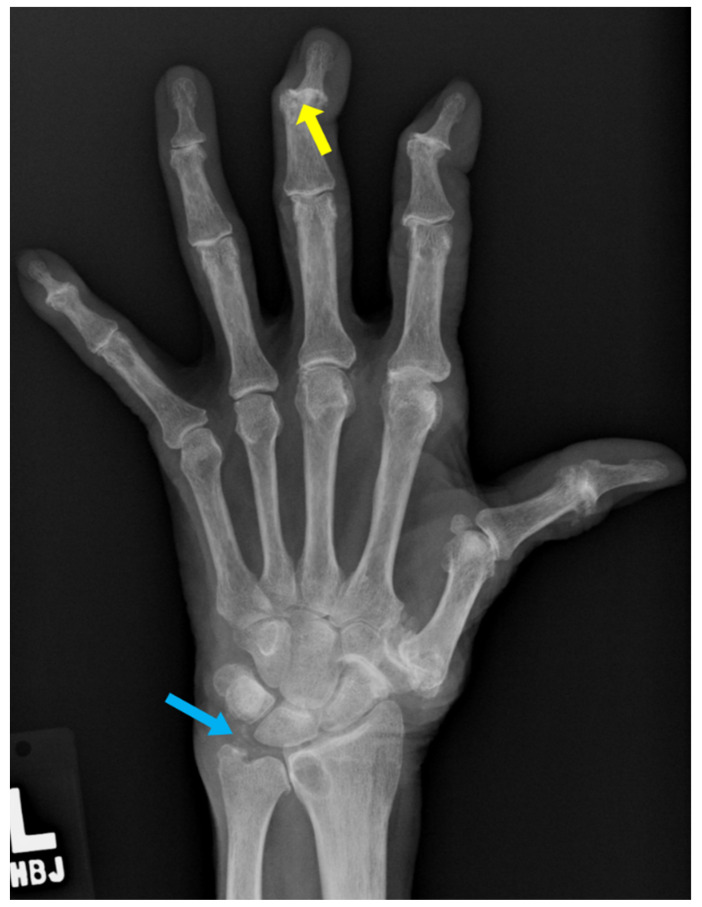
82 year-old-female with hand pain. Chondrocalcinosis (blue arrow). Severe osteoarthritis of the first carpometacarpal and sever joint space narrowing in the second MCP with a small hook osteophyte. Severe osteoarthritis of the DIP joints with central erosions predominantly seen in the middle finger DIP joint. Patient has findings of CPPD arthtopahy and erosive osteoarthritis.

The distribution of degenerative changes in CPPD is different from the primary osteoarthritis and the “wrong” distribution of degenerative changes should raise the possibility of CPPD arthropathy [19]. In addition, cysts are more prominent in CPPD than in osteoarthritis and shoulder and elbow joints are involved unlike osteoarthritis (Figure 10 and Figure 11).

## 8. Gout

Gout results from the deposition of monosodium urate crystals, affects males more frequently than the females and is the most common cause of inflammatory arthritis in men older than 60 years [20]. The prevalence of gout increases in postmenopausal females after the protective effect of estrogen begins to fade away. In primary gout, the uric acid level increases due to inborn error of metabolism. In secondary gout, the level of the uric acid increases by either increased production or decreased excretion due to various diseases. 

The deposition of monosodium urate crystals occurs in tissues with poor blood supply such as cartilage and tendon sheaths and the radiographic findings depends on the location of the tophus. Urate crystals are not radio-opaque; however, depending on the degree of secondary calcium deposition in the tophi, calcifications can be seen with various densities. It takes an average of 7–10 years for radiographic findings of gout to appear, so in this timeframe radiographs are mostly normal or show nonspecific joint effusion or juxta-articular soft tissue swelling [17]. Serum concentration of uric acid can be normal at the time of an acute flare in up to one-third of the patients [21], therefore a normal serum uric acid level does not exclude the diagnosis.

**Figure 12:**
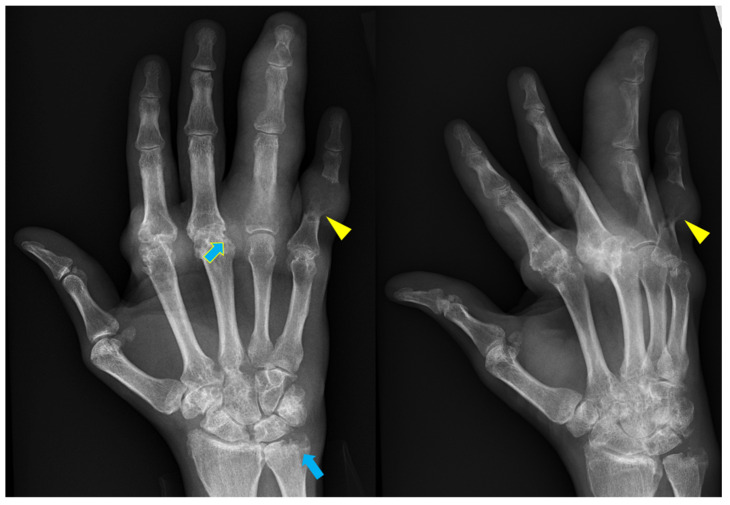
52-year-old male with gout. Mass-like soft tissue densities representing tophi in the second and third MCP joints as well as the ring and fifth fingers with underlying erosive changes and osseous destruction. Sclerosis at the margins of some of the erosions are noted giving the “punched-out” appearance (blue arrows). Overhanging edge is seen in the fifth finger at the margin of the erosion (yellow arrowhead).

The imaging findings of gout in hand radiography include an asymmetrical polyarticular distribution, juxta-articluar eccentric and lobulated soft tissue masses due to tophus deposition, normal mineralization, well-defined erosions with sclerotic margins ( punched-out lesions) and overhanging margins (Figure 12). There is preference of joint involvement in the hands, and erosions can be intra-articular or juxta-articular. As synovitis is not the primary pathophysiology in gout, cartilage damage and joint-space loss are not the primary findings till secondary osteoarthritis develops in the later stages. Extensive erosions from the long-standing soft-tissue tophi can mimic a “mouse or rat bite” appearance [22]. Gouty arthritis can mimic other arthritides and can happen anywhere in the musculoskeletal system so a high index of suspicion should be maintained if the imaging findings are not typical for other types of arthritis.

## 9. Systemic Lupus Erythematous (SLE)

**Figure 13:**
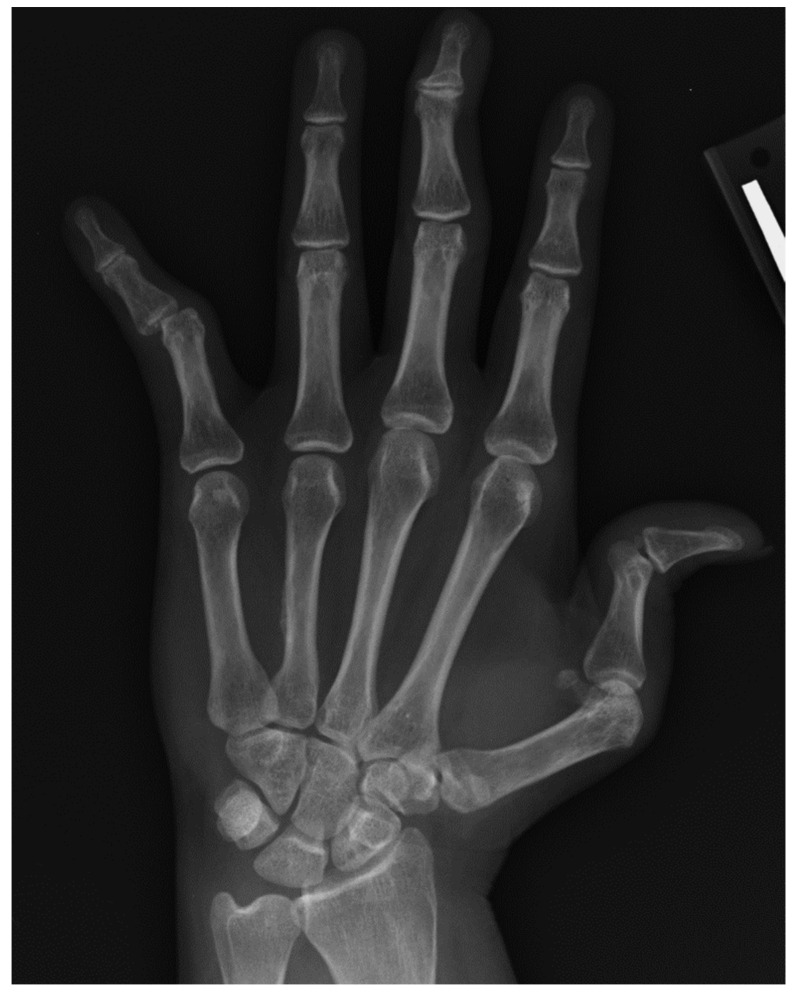
42-year-old female with SLE. Frontal hand radiograph with Boutonnière deformity of the thumb and ulnar subluxation of the fifth finger PIP joint. Note the absence of erosions.

SLE is a systemic autoimmune disease characterized by inflammation of multiple organs. Articular manifestations range from mild and self-limiting arthralgia to persistent arthritis, which can be deforming (i.e., Jaccoud’s arthropathy) and/or erosive (i.e., Rhupus; an entity considered by some clinicians as an overlap syndrome between rheumatoid arthritis and SLE) [23]. The most frequent imaging finding in the hand radiographs is deformity and subluxation without erosions (Figure 13). Except for Rhupus, SLE is considered traditionally as non-erosive [23,24]. Deformities are reducible and alignment improves when the patients’ hands are supported on the radiographic cassette [8]. Other features on radiographs include peri-articular soft-tissue swelling and juxta-articular demineralization. Soft-tissue calcifications are uncommon. 

Jaccoud arthropathy is a non-erosive and deformity arthropathy of the metacarpophalangeal (MCP) and proximal interphalangeal joints, wrists and knees. Hand deformities present as ulnar deviation and subluxation of the MCP similar to RA; however, the deformities are due to ligament laxity and muscle imbalance rather than joint involvement [25].

## 10. Osteoarthritis (OA)

Osteoarthtritis is the most common arthopathy. In primary osteoarthritis, the change in the dynamics and mechanics of a joint results in cartilage loss and bony reactive changes. In secondary osteoarthritis, the cartilage is lost due to the other pathologies such as infection, inflammatory arthritis, gout, CPPD, trauma and hemophilia. Following the loss of cartilage and joint space, the articulating bones come into closer contact and undergo reactive bony changes such as marginal osteophytosis, subchondral cystic changes and sclerosis. Cartilage pieces can be released into the joint space and continue to grow and ossify over time to form osteochondral bodies as joint fluid provides nutrients to the chondrocytes. 

Radiography remains the gold-standard imaging modality to evaluate osteoarthritis [12]. The findings of primary OA in the hands include normal mineralization, osteophyte and cyst formation, and asymmetrical joint space loss. Primary OA involves the distal and proximal interphalangeal joints (DIPs and PIPs) and relatively spares the metacarpophalangeal joints (MCPs). Peri-articular soft-tissue swelling along with osteophyte formation can develop in the PIP and DIP joints, which are known as Bouchard and Heberden nodes, respectively. 

## 11. Systemic Sclerosis

**Figure 14:**
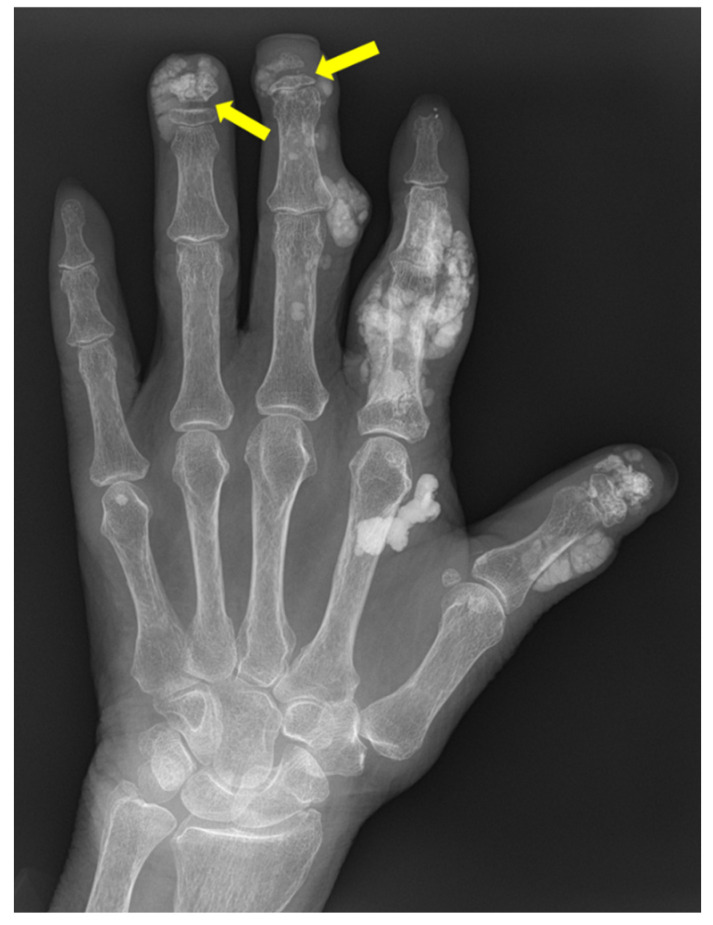
75-year-old female with scleroderma. There is partial osseous loss of the distal tufts of the middle and ring fingers, known as acro-osteolysis (yellow arrows). Soft-tissue calcifications are noted in the thumb, index, middle and ring fingers.

**Figure 15:**
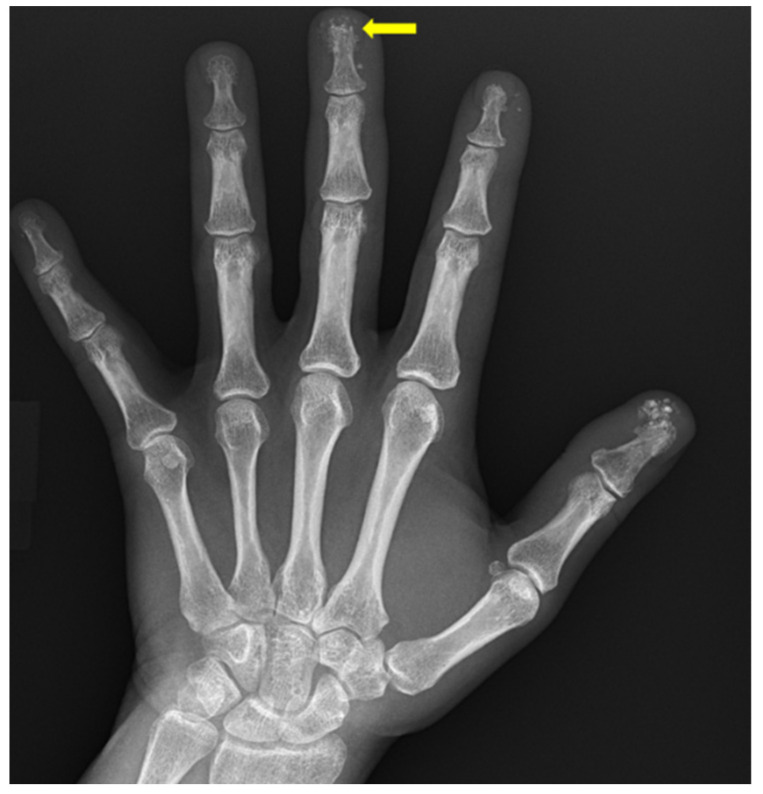
43-year-old female with scleroderma. Early acro-osteolysis at the tip of the middle finger distal tuft (yellow finger). Foci of soft-tissue calcifications in the thumb, index and middle fingers.

Systemic sclerosis, also known as scleroderma, is a multisystem disorder with skin thickening and vasculitis. Acro-osteolysis (osseous loss of the distal phalanges of the hands and feet) and soft-tissue calcifications are the main radiographic findings. Acro-osteolysis is seen in approximately 40–80% of the cases and manifests as resorption of the tufts, penciling, and resorption of the entire distal phalanx [8]. Other main differential diagnoses of acro-osteolysis include hyperparathyroidism, psoriatic arthritis, and thermal injuries. Arthritis is uncommon in early stages of the disease and might develop later on as erosions and cartilage loss. Digital soft-tissue calcification is seen in 10–30% of patients with systemic sclerosis [8] (Figure 14 and Figure 15).

Other conditions mimicking inflammatory arthritis:

## 12. Septic Arthritis

**Figure 16:**
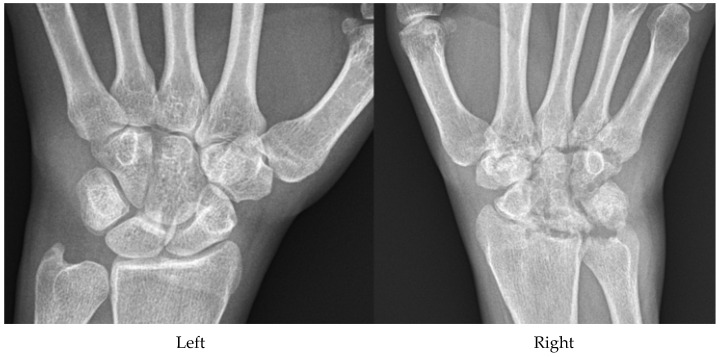
37-year-old female with septic arthritis of the right wrist. Patient presented with pain and swelling in the right wrist for a few weeks along with fever and chills. Radiographs show erosive changes in the right wrist with soft-tissue swelling. No other joints are involved. Septic arthritis is the diagnosis of exclusion in all patients presenting with monoarthritis and should not be overlooked.

Septic arthritis is an important consideration when only one joint is involved (monoarthritis). Infection of a joint can be the result of direct extension of infection from subjacent soft tissues (e.g., ulcers in diabetes, decubitus ulcer or penetrating injuries) or secondary to hematogenous spread of pathogens. Radiographic findings include soft-tissue swelling and joint effusion in early stages. Joint-space narrowing and erosive changes are appreciated in later stages as the cartilage is destructed due to synovitis and infection. It is important to diagnose septic arthritis in the early stages, as cartilage and joint destruction can result in osteoarthritis in untreated cases. In the cases of chronic septic arthritis, indolent infections such as tuberculosis should be contemplated [26]. Septic arthritis is a diagnosis of exclusion and should be considered in every case of monoarthritis unless proven otherwise (Figure 16). 

## 13. Conclusions

Radiography of the hands is a commonly available and cost-effective imaging modality that can provide invaluable information about the pattern of joint involvement and should be the first imaging modality of choice in patients presenting with hand and wrist pain.

## Figures and Tables

**Table 1:** Summary of imaging findings of different arthritis in the hand radiography.

Disease	Rheumatoid Arthritis	Psoriasis	Erosive OA	CPPD	Gout	SLE
**Joints**	MCP > IP DIP unusual	IP > MCP	IP > MCP (mostly DIP)	MCP Radiocarpal	Any	Typically MCPs
**Osteopenia**	Juxta-articular then diffuse	No		Not primarily	No	Juxta-articular
**Erosions**	Marginal	Marginal and then central	Central	Not dominant	Well-defined with sclerotic margins	Typically absent
**Periostitis**	No periostitis	Periostitis				
**Distribution**	Polyarticular	2/3 asymmetric and mono-oligoarticular	Polyarticular Bilateral Usually symmetric	MCP Radiocarpal	Asymmetrical polyarticular	Polyarticular
**Hallmarks**		Sausage-digits Micky-mouse Pencil-in-cup. No osteophyte formation	Gull-wing (not specific, also can be seen in RA and PsA)	More prominent cysts Wrong distribution for primary OA Elbow and shoulders involved Chondrocalcinosis involving the triangular fibrocartilage complex (TFCC), scapholunate and lunotriquetral ligaments	Punched out erosions with overhanging edges	Reducible deformity and subluxations Non-erosive
